# Association between childhood trauma and risk for obesity: a putative neurocognitive developmental pathway

**DOI:** 10.1186/s12916-020-01743-2

**Published:** 2020-10-15

**Authors:** Qiang Luo, Lingli Zhang, Chu-Chung Huang, Yan Zheng, Jonathan W. Kanen, Qi Zhao, Ye Yao, Erin B. Quinlan, Tianye Jia, Tobias Banaschewski, Arun L. W. Bokde, Uli Bromberg, Christian Büchel, Herta Flor, Vincent Frouin, Hugh Garavan, Penny Gowland, Andreas Heinz, Bernd Ittermann, Jean-Luc Martinot, Marie-Laure Paillère Martinot, Frauke Nees, Dimitri Papadopoulos Orfanos, Luise Poustka, Sarah Hohmann, Juliane H. Fröhner, Michael N. Smolka, Henrik Walter, Robert Whelan, Barbara J. Sahakian, Gunter Schumann, Fei Li, Jianfeng Feng, Sylvane Desrivières, Trevor W. Robbins, for the IMAGEN consortium

**Affiliations:** 1 Institute of Science and Technology for Brain-Inspired Intelligence, Ministry of Education Key Laboratory of Computational Neuroscience and Brain-Inspired Intelligence, Fudan University, Shanghai, 200433 People’s Republic of China; 2 Developmental and Behavioral Pediatric Department & Child Primary Care Department, Ministry of Education Key Laboratory for Children’s Environmental Health, Xinhua Hospital, Shanghai Jiao Tong University School of Medicine, Shanghai, 200092 People’s Republic of China; 3 State Key Laboratory of Medical Neurobiology and Ministry of Education Frontiers Center for Brain Science, Institutes of Brain Science and Human Phenome Institute, Fudan University, Shanghai, 200433 People’s Republic of China; 4 State Key Laboratory of Genetic Engineering, School of Life Sciences, Human Phenome Institute, Zhongshan Hospital, Fudan University, 2005 Songhu Road, Shanghai, 200438 People’s Republic of China; 5 Departments of Psychiatry and Psychology and the Behavioural and Clinical Neuroscience Institute, University of Cambridge, Cambridge, CB2 3EB UK; 6 Medical Research Council - Social, Genetic and Developmental Psychiatry Centre, Institute of Psychiatry, Psychology and Neuroscience, King’s College London, London, SE5 8AF UK; 7 Department of Child and Adolescent Psychiatry and Psychotherapy, Central Institute of Mental Health, Medical Faculty Mannheim, Heidelberg University, Square J5, Mannheim, Germany; 8 Discipline of Psychiatry, School of Medicine and Trinity College Institute of Neuroscience, Trinity College Dublin, Dublin, Ireland; 9 University Medical Centre Hamburg-Eppendorf, Hamburg, Germany; 10 Department of Cognitive and Clinical Neuroscience, Central Institute of Mental Health, Medical Faculty Mannheim, Heidelberg University, Mannheim, Germany; 11 Department of Psychology, School of Social Sciences, University of Mannheim, Mannheim, Germany; 12 NeuroSpin, Commissariat à L’énergie Atomique, Université Paris-Saclay, Gif-sur-Yvette, France; 13 Departments of Psychiatry and Psychology, University of Vermont, Burlington, USA; 14 Sir Peter Mansfield Imaging Centre School of Physics and Astronomy, University of Nottingham, University Park, Nottingham, UK; 15 Department of Psychiatry and Psychotherapy, Campus Charité Mitte, Charité, Universitätsmedizin Berlin, Berlin, Germany; 16 Physikalisch-Technische Bundesanstalt (PTB), Abbestr. 2-12, Berlin, Germany; 17 Institute National de la Santé et de la Recherche Médicale Unit 1000, Neuroimaging and Psychiatry, University Paris Sud–Paris Saclay, University Paris Descartes, Paris, France; 18 Service Hospitalier Frédéric Joliot, Orsay, France; 19 Maison de Solenn, Paris, France; 20 Assistance Publique–Hôpitaux de Paris, Department of Child and Adolescent Psychiatry, Pitié-Salpêtrière Hospital, Paris, France; 21 Department of Child and Adolescent Psychiatry and Psychotherapy, University Medical Centre Göttingen, Göttingen, Germany; 22 Clinic for Child and Adolescent Psychiatry, Medical University of Vienna, Währinger Gürtel, Vienna, Austria; 23 Department of Psychiatry and Neuroimaging Center, Technische Universität Dresden, Dresden, Germany; 24 School of Psychology and Global Brain Health Institute, Trinity College Dublin, Dublin, Ireland; 25 Department of Computer Science, University of Warwick, Coventry, UK; 26 Collaborative Innovation Center for Brain Science, Fudan University, Shanghai, People’s Republic of China

**Keywords:** Childhood trauma, Adult obesity, Neurocognitive control pathway, Structural brain imaging

## Abstract

**Background:**

Childhood trauma increases the risk for adult obesity through multiple complex pathways, and the neural substrates are yet to be determined.

**Methods:**

Participants from three population-based neuroimaging cohorts, including the IMAGEN cohort, the UK Biobank (UKB), and the Human Connectome Project (HCP), were recruited. Voxel-based morphometry analysis of both childhood trauma and body mass index (BMI) was performed in the longitudinal IMAGEN cohort; validation of the findings was performed in the UKB. White-matter connectivity analysis was conducted to study the structural connectivity between the identified brain region and subdivisions of the hypothalamus in the HCP.

**Results:**

In IMAGEN, a smaller frontopolar cortex (FPC) was associated with both childhood abuse (CA) (*β* = − .568, 95%CI − .942 to − .194; *p* = .003) and higher BMI (*β* = − .086, 95%CI − .128 to − .043; *p* < .001) in male participants, and these findings were validated in UKB. Across seven data collection sites, a stronger negative CA-FPC association was correlated with a higher positive CA-BMI association (*β* = − 1.033, 95%CI − 1.762 to − .305; *p* = .015). Using 7-T diffusion tensor imaging data (*n* = 156), we found that FPC was the third most connected cortical area with the hypothalamus, especially the lateral hypothalamus. A smaller FPC at age 14 contributed to higher BMI at age 19 in those male participants with a history of CA, and the CA-FPC interaction enabled a model at age 14 to account for some future weight gain during a 5-year follow-up (variance explained 5.8%).

**Conclusions:**

The findings highlight that a malfunctioning, top-down cognitive or behavioral control system, independent of genetic predisposition, putatively contributes to excessive weight gain in a particularly vulnerable population, and may inform treatment approaches.

## Background

Overweight and obesity affect one third of adults in developed countries [1], and life expectancy could be shortened by 2–4 years for those who became obese [2]. Obesity interventions are effective in the short term, but subsequent weight regain often occurs: long-term weight management is a primary treatment challenge [3]. Identifying the contributors to long-term weight gain will be essential for combating the obesity epidemic [4, 5].

Long-term weight management involves more than detecting physiological signals about hunger or satiety: cognitive control is required to resist urges to eat and helps avoid or shift attention away from food cues in the environment or retrieved from memory [6]. The hypothalamus is a center for eating behavior, while the prefrontal cortex is critical for cognitive control: Given their reciprocal connections, observed in animal models [7–9], early-life damage to this neurocognitive system may impair both motivation and capability for long-term weight management [10]. For example, a 2017 study has demonstrated that compared with the controls, a chronic early-life stress (ES; between P2 and P9) mouse model actually had reduced mRNA expression of leptin lasting to adulthood and both reduced total body fat mass and impaired learning and memory in adulthood; however, compared with the control mice, the mice exposed to a moderate western-style diet showed higher body fat accumulation at P98 as compared to P42 [11]. However, this has been difficult to test in humans using randomized clinical trials. In observational studies, childhood trauma has been identified as a key environmental risk factor that can disrupt brain development [12, 13] and increase risk for obesity [14, 15]. We therefore harnessed a population-based longitudinal neuroimaging cohort in an effort to identify a neurocognitive control (NcC) pathway relating to childhood trauma and the risk for obesity.

Human studies have begun to reveal enduring effects of childhood trauma on neural systems [12], including reduced gray matter volume (GMV) in the prefrontal cortex [16] and ventral striatum [17]; however, neural contributions to an NcC pathway remain to be determined. Neural changes after childhood trauma are additionally modulated by sex [18]: brain development is sex-dimorphic [19] and an NcC pathway may be sex-dimorphic as well. Obesity itself can affect the brain [20, 21]: evidence of neural changes temporarily preceding excessive weight gain would further strengthen the case for an NcC pathway. To address these questions, we analyzed data from the IMAGEN longitudinal neuroimaging study of adolescents [22].

We hypothesized that some structural changes in the brain might link the childhood trauma to higher body mass index (BMI). To identify the candidate brain structures to serve as this link, we first conducted a whole-brain voxel-wise association study (BWAS) of GMV to identify overlapping neuroanatomical correlates between childhood trauma and higher body mass index (BMI) using the IMAGEN sample. To account for the sex-dimorphic in brain development, we conducted this BWAS for females and males separately. To ensure specificity of our finding, we accounted for potential confounding factors: polygenetic risk for obesity [23], family socioeconomical status [24], changes due to illegal drug use [25], and elevated depressive symptoms [26]. To validate and extend our results to a wider age range, we sought to replicate our findings using an independent sample with a mean age of 56.89 years (UK Biobank [27]). Next, we mapped white-matter connectivity between the hypothalamus and the cortical areas, to explore the potential structural basis for supporting cognitive control over eating behavior mediated by the hypothalamus. Given that the hypothalamus is a small structure (~ 1 cm^3^), we analyzed the 7-Tesla (7-T) diffusion tensor imaging (DTI) data from the Human Connectome Project [28] with a high spatial resolution (1.5 × 1.5 × 1.5 mm^3^). Finally, to understand the directionality of the identified associations, we conducted a longitudinal analysis in the IMAGEN cohort to test whether neural correlates of childhood trauma at age 14 preceded excessive weight gain during a 5-year follow-up period.

## Methods

### Participants

#### Discovery sample

IMAGEN is a multicenter longitudinal study of healthy youths in Europe [22]. The database we used was released in June 2016. Briefly, 2087 participants were recruited at age 14, and among them, 1650 returned for follow-up at age 19. The inclusion criteria included (1) participants with childhood maltreatment assessment, (2) with BMI information at both time points, and (3) structural images at both time points. The exclusion criterion was participants who were underweight (BMI < 18.5 kg/m^2^) at follow-up. After data quality controls in all these domains—neuroimaging (*n* = 949) and behavioral assessments (trauma: *n* = 1159, and BMI: *n* = 1042) at both baseline and follow-up, 639 young adults (325 females) with a mean (SD) age of 19.06 (.70) were included in the current study. Of these, 557 adolescents (278 females) had genetic information (Table 1, Additional file 1: Method S1 [22, 29–45]).

**Table 1:** Demographic characteristics of 639 young participants from the IMAGEN study ^a^

**Participants**	**Young males**		**Young females**	
	**No exposure**	**Exposure**	**No exposure**	**Exposure**
Childhood Abuse	240	74	240	85
EA	251	63	254	71
PA	298	16	302	23
SA	305	9	302	23
Childhood Neglect	202	112	237	88
EN	227	87	261	64
PN	263	51	281	44
	**No childhood abuse**	**Childhood abuse**	**No childhood abuse**	**Childhood Abuse**
Baseline BMI	20.76 ± 3.16	21.91 ± 3.87	21.26 ± 3.25	21.68 ± 3.19
Follow-up BMI	23.29 ± 3.52	24.79 ± 4.87	23.27 ± 3.87	23.98 ± 4.96
PRS_BMI_	− 1.760 × 10^−4^ (4.442 × 10^−5^)	− 1.645 × 10^−4^ (3.891 × 10^−5^)	− 1.782 × 10^−4^ (4.547 × 10^−5^)	− 1.661 × 10^−4^ (4.117 × 10^−5^)
Illegal drug use	50.25%	67.21%	35.61%	47.95%
Depressive score	19.19 ± 1.52	18.46 ± 2.09	18.93 ± 1.85	17.53 ± 2.74

Numbers of subjects with a particular characteristic are listed as integers or percentage, and quantitative measurements are presented as mean values ± standard deviations

*EA* emotional abuse, *PA* physical abuse, *SA* sexual abuse, *EN* emotional neglect, *PN* physical neglect, *BMI* body mass index, *PRS*_*BMI*_ polygenic risk score for obesity

#### Validation sample

UK Biobank (UKB) is a large population-based cohort study of adults in the UK. After quality controls for neuroimaging data and behavioral assessments, 4121 participants (2396 females) with a mean (SD) age of 56.89 (5.02) years were included (Additional file 1: Method S2 and Table S1).

#### White-matter connectivity sample

Human Connectivity Project (HCP 1200 Subject Release; Last Updated: April 2018) from the Washington University in St. Louis–University of Minnesota (WU-Minn HCP) Consortium provided 178 participants (109 females; mean [SD] age of 29.5 [3.33] years) with 7-T diffusion magnetic resonance imaging (dMRI) after preprocessing [39–41].

### Measurements

#### Anthropometric indices

Weight and height were measured to calculate BMI [weight(kg)/height(m)^2^]. Overweight was defined as 30 kg/m^2^ > BMI ≥ 25 kg/m^2^, obesity as BMI ≥ 30 kg/m^2^.

#### Childhood trauma

The well-established Childhood Trauma Questionnaire (CTQ [29, 46–48]) was used to assess the history of childhood trauma before the age of 19 in IMAGEN, including abuse (emotional, physical, and sexual) and neglect (emotional and physical). Since the participants in the IMAGEN study were healthy subjects, we used the lowest cut-off as 8 for EA, 7 for PA, 5 for SA, 9 for EN, and 7 for PN [29]. If any type of abuse/neglect occurred, abuse/neglect was scored as “1”; if not, a score of “0” was recorded (Additional file 1: Method S1). In UKB, the following questions were asked: physically abused by family member as a child (Field ID: 20488), felt hated by family members as a child (Field ID: 20487), and sexually molested as a child (Field ID: 20490) (Additional file 1: Method S2).

#### Neuroimaging data

In IMAGEN, T1-weighted images were collected using 3-T scanners and the ADNI protocols [33]. UKB used a Siemens Skyra 3-T scanner with a 3D MPRAGE protocol (Additional file 1: Method S2). All data were preprocessed in SPM8 using the VBM8 toolbox, including the segmentation, normalization, modulation, and smoothing. The resulting voxel size was 1.5 × 1.5 × 1.5 mm^3^ (Additional file 1: Method S1)*.* HCP used a Siemens 7-T MAGNETOM scanner with a high spatial resolution of 1.05 mm isotropic (Additional file 1: Method S3) [49].

#### Polygenic risk score

Polygenic risk scores (PRSs) for higher BMI (PRS_BMI_) were calculated using the PRSice software (http://prsice.info/) [35]. To generate PRS_BMI_ in IMAGEN, we used GWAS summary data from GIANT, which included 2,554,637 SNPs and up to 339,224 individuals of European ancestry [36] (Additional file 1: Method S1).

### Statistical analysis

#### Behavioral association analysis

We applied linear regression models to evaluate the relationships between childhood trauma and BMI measured at age 19 in male and female separately, controlling for data collecting site. The false discovery rate (FDR) correction was employed to control for multiple comparisons. Then in the sensitivity analysis, we tested the confounding effects of PRS_BMI_, family socioeconomic status, stressful life events in the past year, birth weight, depressive symptoms, and illegal drug use (Additional file 1: Method S1). Statistical analyses were performed using IBM SPSS Statistics, Version 22. The coefficient (unstandardized, *β*) of the linear regression models and its 95% confidence interval (CI) were reported.

#### Structural association analysis

We conducted a voxel-wise association study of GMV with either childhood abuse, PRS_BMI,_ or BMI at age 19 years, in the female and male participants, separately. We focused our analysis on gray matter (i.e., 380,537 voxels defined by the automatic anatomic labeling atlas [50]) and considered the following covariates: data collecting site, handedness, and total intracranial volume (TIV). We identified significant clusters by permutation-based TFCE (Threshold-Free Cluster Enhancement, 5000 permutations, no acceleration method, *p* < .05, 2-tailed) [51] with cluster size greater than 217 voxels (approximately 4/3 × π × [3.3970 × 1.645]^3^/1.5^3^ voxels falling into the 90% confidence interval of the smoothing kernel [33]). We defined the overlapping clusters that were associated with both CA and BMI as the abuse ROIs. In the following analyses, we focused on the gray matter volumes of the abuse ROIs. To test whether stronger abuse-brain association was correlated with stronger abuse-BMI association, we calculated the correlation between these two associations across multiple sites of data collection.

#### White-matter connectivity analysis

The pre-processed 7-T dMRI data were downloaded from the ConnectomeDB, which is a data sharing platform provided by HCP (http://db.humanconnectome.org) [52]. We used state-of-the-art brain atlases, including HCP-MMP (multi-modal parcellation) for cortical areas [44] and CIT168 (California Institute of Technology) for the hypothalamus [45]. The cortical connectivity of the hypothalamus (HTH; 8 subdivisions defined by lateral-medial and anterior-posterior; Additional file 1: Figure S1) was counted as the number of reconstructed tracks ending in each cortical area using DSI Studio (http://dsi-studio.labsolver.org). Combining one-sample sign test with false discovery rate, we assessed whether the median of the fiber number (FN) estimated for one brain area was greater than the randomly assigned FN according to the size of this area (Additional file 1: Method S3).

#### Mediation analysis

We tested the mediation effect of the FPC volume at age 19 on the association between childhood abuse and BMI at age 19, while controlling for data collecting site, handedness, TIV, and PRS_BMI_. We applied a bootstrap procedure provided by PROCESS in SPSS (http://www.processmacro.org/) to test the mediation (indirect) effect. A significant indirect effect was identified when the bootstrap confidence interval did not include zero.

#### Cross-lagged panel analysis

To determine the directionality of the relationship between the GMV of the abuse ROI identified above and the BMI in the group with childhood abuse, we employed a two-wave cross-lagged panel model (CLPM) [53] using the data collected at ages 14 and 19 with the Mplus Version 7. The FDR was applied to correct for multiple comparisons between the cross-lagged coefficients of the BMI➔GMV and the GMV➔BMI directions. Furthermore, we compared this longitudinal association between the groups with and without childhood abuse by the multi-group analysis of CLPM. We considered the following covariates: data collecting site, handedness, baseline TIV, PRS_BMI_, and family socioeconomic status. Significance levels were given by 5000 bootstraps.

## Results

### Childhood abuse associated with higher BMI independent of genetic risk

**Fig. 1:**
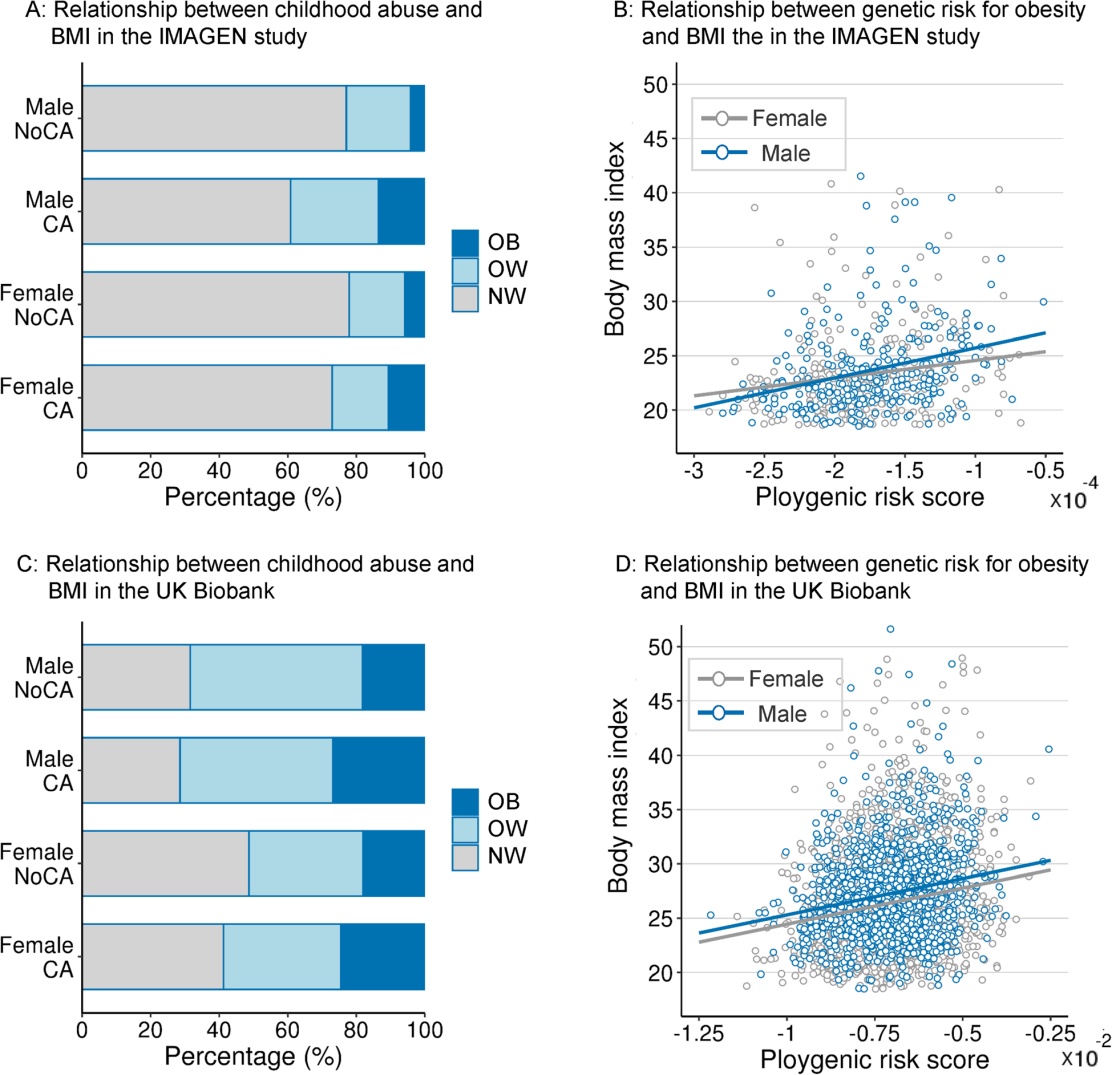
Association between childhood abuse, PRS_BMI_, and BMI. **a** Childhood abuse associated with higher rate of overweight and obesity in male participants, but not in female participants in the IMAGEN study. **b** Polygenic risk for obesity associated with BMI’s in both male and female participants in the IMAGEN study. **c** Childhood abuse associated with higher BMI in both male and female participants in the UK Biobank. **d** Polygenic risk for obesity associated with BMI in both male and female participants in the UK Biobank. NoCA, no childhood abuse; CA, childhood abuse; OB, obesity; OW, overweight; NW, normal weight

For IMAGEN, we found that young adults with a history of childhood abuse (CA) had higher BMI compared with those without (*β* = 1.018, 95%CI .292 to 1.745; *p* = .006). This effect was significant in male participants (*β* = 1.445, 95%CI .418 to 2.471; *p* = .006, FDR-*p* = .012; Fig. 1a), but not in female participants (*β* = .543, 95%CI − .491 to 1.577, *p* = .30). The PRS_BMI_ was correlated with BMI in both sexes (male: *β* = 1.237, 95%CI .783 to 1.690; *p* < .001; female: *β* = .785, 95%CI .303 to 1.268; *p* = .001; Fig. 1b). Moreover, this association in male participants remained significant after controlling for both PRS_BMI_ (*β* = 1.119, 95%CI .068 to 2.170; *p* = .037) and other possible confounders, such as family socioeconomic status, stressful life events in the past year, birth weight, depressive symptoms, and illegal drug use (Additional file 1: Table S2). The PRS_BMI_-by-CA interaction effect on BMI in the male participants did not reach statistical significance (*p* = 0.321).

### Smaller frontopolar cortex associated with both childhood abuse and higher BMI

**Fig. 2:**
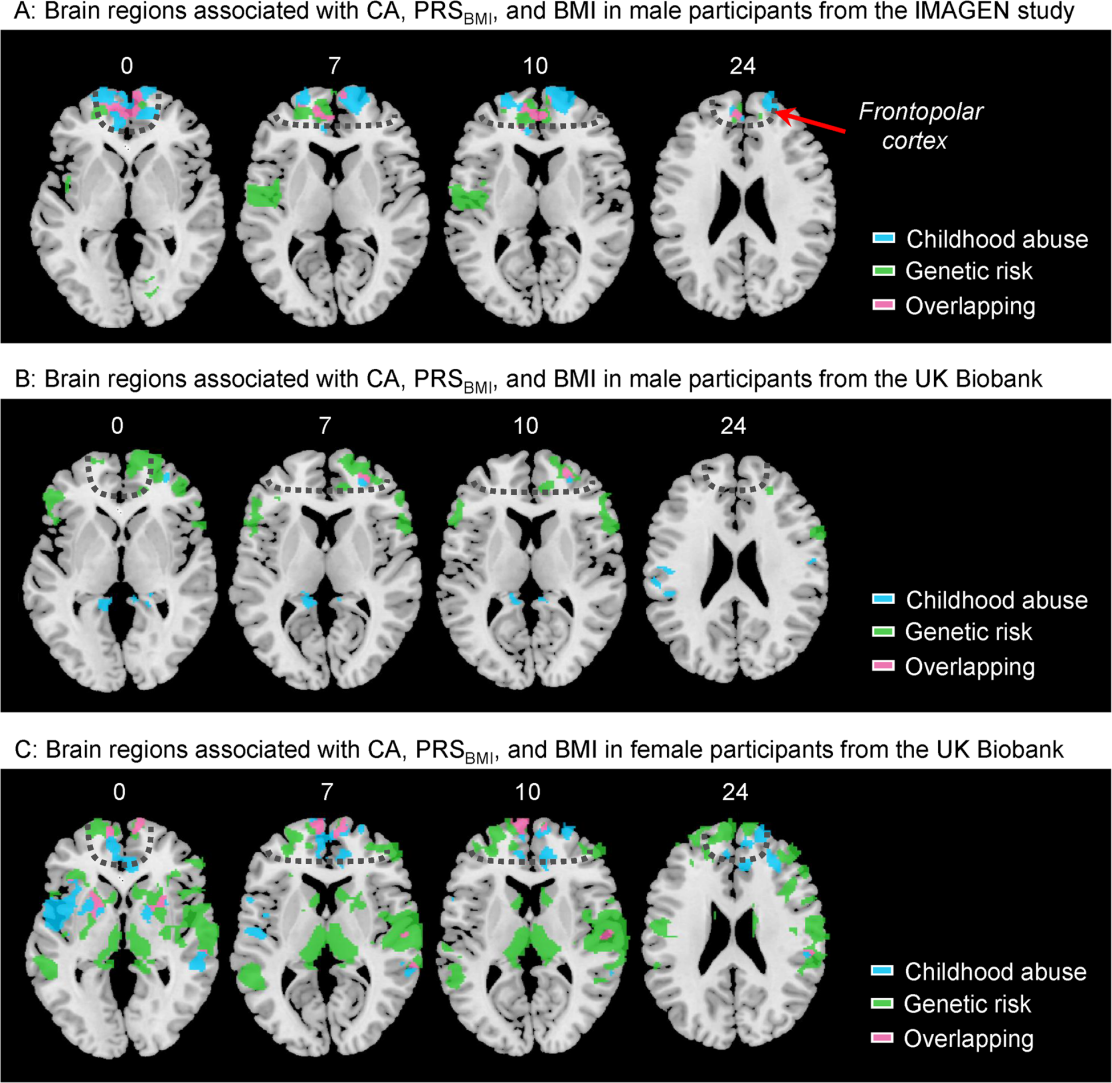
Brain regions associated with childhood trauma, genetic risk for higher BMI, and BMI. **a** The significant brain regions with GMV associated with both childhood abuse and BMI (marked by blue and pink), associated with both genetic risk and BMI (marked by green and pink), and the overlapped brain regions (i.e., brain regions associated with childhood abuse, genetic risk, and BMI, marked by pink) in male participants from the IMAGEN study. **b**, **c** The significant brain regions with GMV associated with variables mentioned above in male or female participants from the UK Biobank. Gray dotted line roughly marked the inner contour of the frontopolar cortex. GMV, gray matter volume; CA, childhood abuse; BMI, body mass index; PRS_BMI_, polygenic risk score for obesity

**Fig. 3:**
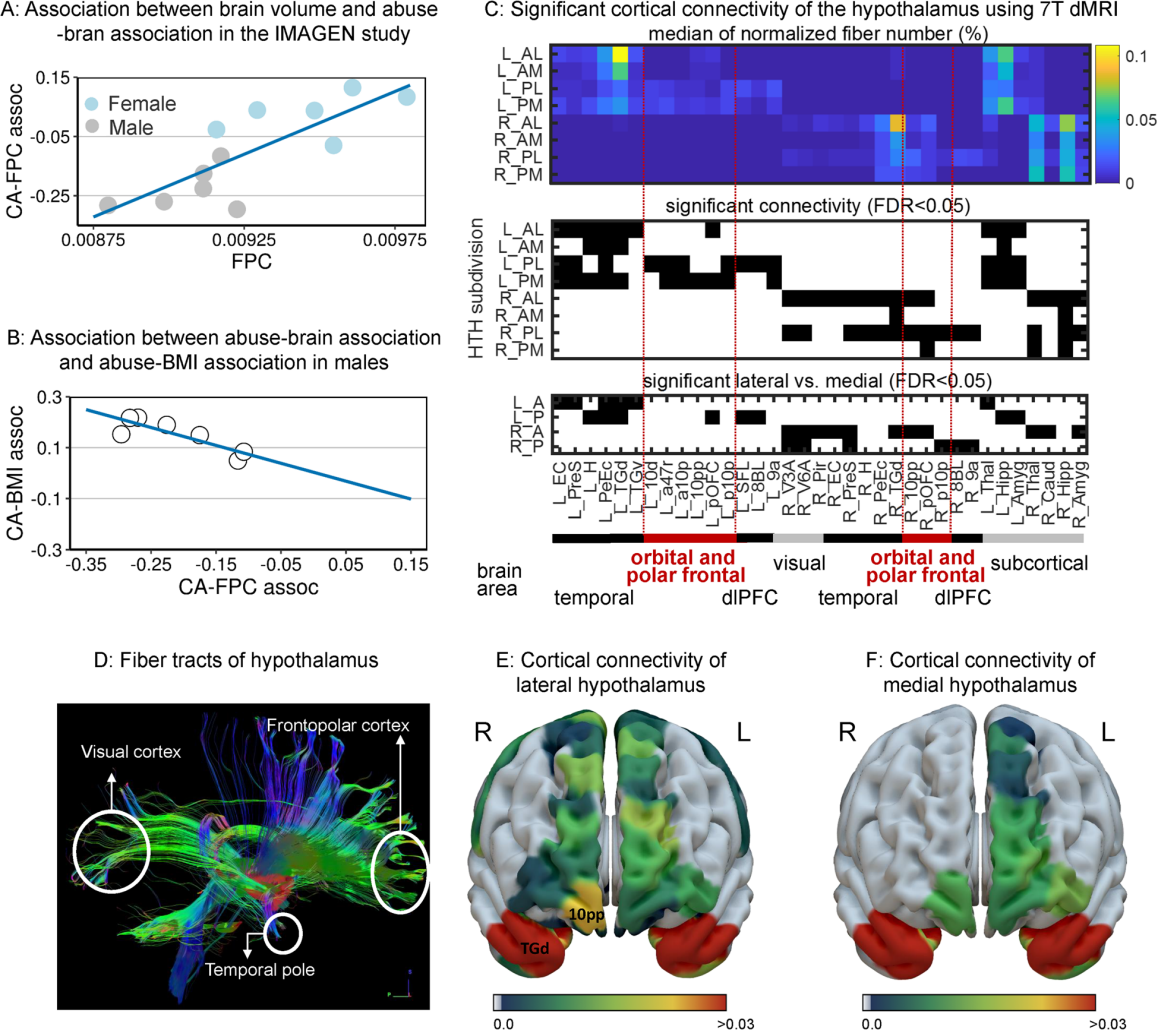
Associations were coupled between abuse-BMI and abuse-brain. **a** Smaller volume of the FPC was associated with deeper negative association between CA and the FPC volume (CA-FPC assoc) across both male (gray dots) and female (blue dots) subjects at the 6 data collection sites in the IMAGEN cohort. The FPC volume shown here was the ratio between the raw FPC volume and the TIV. **b** The partial correlation coefficient between CA and BMI was associated with the partial correlation coefficient between CA and the FPC volume in male subjects across the 6 data collection sites in the IMAGEN study and the UK Biobank sample. **c** Significant cortical connectivity of the hypothalamus using 7-T dMRI data from HCP. The fiber number was normalized as a percentage of the connections tracked between one brain region and one subdivision of the hypothalamus among all its tracked connections. The connections survived the FDR correction were reported. The upper plot shows the median of the fiber number, the middle plot marks the significant connectivity in black, and the lower plot shows the significant differences in connectivity between lateral and medial hypothalamus. The abbreviations of the cortical parcels are defined by the HCP-MMP atlas. The hypothalamus was divided into 8 subdivisions along the anterior-posterior and the medial-lateral lines. L, left; R, right; AL, anterior lateral; AM, anterior medial; PL, posterior lateral; PM, posterior medial. **d** One exemplar of the fiber tracking results. Only the fibers ending in the hypothalamus (marked in red, defined by the CIT168 atlas) were shown. The surface views of the white matter connectivity of the lateral (**e**) and the medial (**f**) hypothalamus. The maximum of the connectivity (normalized fiber number, %) between the anterior and posterior parts of either the lateral or the medial hypothalamus. FPC, frontopolar cortex; CA, childhood abuse; TIV, total intracranial volume; BMI, body mass index; HTH, hypothalamus

We first confirmed a previous finding [54] that lower GMV of a frontopolar cluster was associated with both greater PRS_BMI_ and higher BMI in male participants from IMAGEN (Additional file 1: Table S3). This demonstrated that the brain volume associations identified in this manner are replicable. Using the same approach, we then identified that lower GMV of a frontal cluster was associated with CA (Additional file 1: Table S3). The abuse-cluster overlapped with the BMI-cluster in the FPC (Fig. 2a). No significant neuroanatomical association was identified for CA in the female brains in IMAGEN. The association between childhood abuse and smaller FPC volume in male participants remained significant after controlling for both PRS_BMI_ and other possible confounders (Additional file 1: Table S4). The FPC volume was smaller in male than in female participants at ages 19 (*β* = − .431, 95%CI − .660 to − .203, *p* < .001), and a significant interaction effect was observed between CA and sex on the volume of the FPC (*β* = − .824, 95%CI − 1.351 to − .296, *p* = .002). We found that smaller FPC was correlated with greater negative abuse-FPC association across the 6 data collection sites of IMAGEN (*β* = .002, 95%CI .001 to .002; *p* = .002; Fig. 3a). FPC volume was not associated with depressive symptoms (*n* = 285; *r* = .05; *p* = .4) or lifetime illegal drug use (*n* = 264; *r* = − .11; *p* = .1 after controlling for BMI).

### Replication using a larger cohort

In UKB, we found that CA was associated with higher BMI in both sexes after controlling for PRS_BMI_ (male: *β* = .790, 95%CI .317 to 1.263; *p* = .001; female: *β* = 1.039, 95%CI .564 to 1.514; *p* < .001; Fig. 1c, d), and the effect size of the trauma-BMI association remained at a comparable level between the IMAGEN and UKB cohorts (.32 kg/m^2^ in males from IMAGEN and .17 kg/m^2^ in males from UKB; Additional file 1: Table S5). Regardless of age-related effects on the brain [55], we were able to confirm that smaller FPC was associated with both CA and higher BMI in male participants (*n* = 1725) from UKB (Fig. 2b; Additional file 1: Table S6). Consistent with the behavioral association, we identified that an FPC cluster with lower GMV was associated with both CA and higher BMI in female participants (*n* = 2396) from UKB (Fig. 2c; Additional file 1: Table S7). Considering PRS_BMI_ as an additional covariate, these volumes were still associated with CA (male: *β* = − .194, 95%CI − .308 to − .081; *p* = .001; female: *β* = − .164, 95%CI − .255 to − .073; *p* < .001; controlling for BMI) and BMI (male: *β* = − .044, 95%CI − .057 to − .031; *p* < .001; female: *β* = − .064, 95%CI − .073 to − .055; *p* < .001; controlling for CA).

### Stronger abuse-BMI association coupled with stronger abuse-brain association

Across the 6 data collection sites in IMAGEN and the UKB cohort, we found that greater negative abuse-brain association was correlated with a stronger positive abuse-BMI association in male participants (*β* = − 1.033, 95%CI − 1.762 to − .305; *p* = .015; Fig. 3b), but such coupling weakened in female participants (*z* = − 1.94, *p*_1-tailed_ = .03; Additional file 1: Figure S2).

### Frontopolar cortex is structurally connected with a hypothalamic center of eating behavior

Using dMRI data from HCP (*n* = 156), we examined the structural connectivity between each of the 8 subdivisions of the hypothalamus (i.e., divided using the anterior-posterior parts by the lateral-medial parts in both hemispheres; Additional file 1: Figure S1) and 374 brain regions (i.e., 360 cortical regions and 14 subcortical regions). We found 28 cortical areas and 7 subcortical areas had significant white matter connections with the hypothalamus after FDR correction (Fig. 3c, d; Additional file 2). The strongest connectivity was between the left dorsal temporal gyrus (L_TGd) and the left anterior lateral hypothalamus (L_AL; median of the normalized fiber number: 10%; FDR-*p* < .001; Fig. 3e). The most polar subdivision of Brodmann area (BA) 10 in the right hemisphere (R_10pp), ranked as the third most connected cortical areas, had significant connections with both the posterior (2.2%; FDR-p < .001) and anterior (1.0%; FDR-p < .001) lateral hypothalamus (Fig. 3e). At the right anterior hypothalamus, R_10pp was connected much stronger to the lateral hypothalamus compared with the medial hypothalamus (FDR-*p* = .009; Fig. 3f; Additional file 1: Figure S3).

### From cross-sectional association to longitudinal prediction

**Fig. 4:**
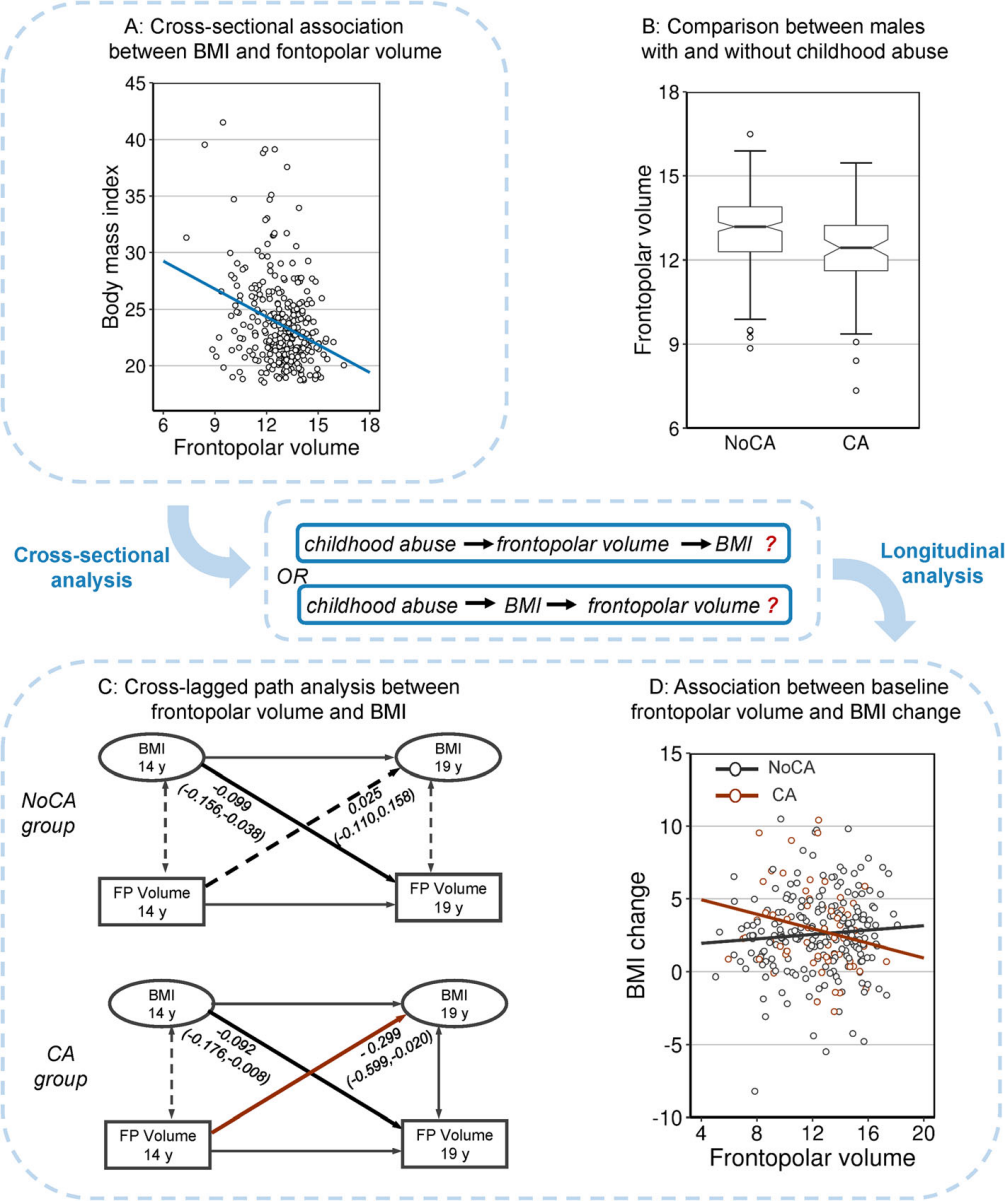
Associations among frontopolar volumes, childhood trauma, and BMI in the IMAGEN study. **a**-**b** The associations of the frontopolar volume with both childhood abuse and BMI in male participants at age 19 years. The frontopolar volumes were identified as the GMVs of brain region associated with both childhood abuse and BMI (marked by blue and red in Fig. 2a). These cross-sectional analyses indicated two potential paths among childhood abuse, frontopolar volume, and BMI. **c** Cross-lagged panel analysis between frontopolar volume and BMI. In males who did not experience childhood abuse, baseline BMI was significantly associated with frontopolar volume at follow-up (up); while in males who did experience childhood abuse, paths existed at both directions (down). **d** Association between BMI at age 14 years and frontopolar volumetric change between age 14 and 19; these analyses based on longitudinal designs indicated the interaction between the frontopolar volume at age 14 years and childhood abuse contributed to explaining weight gain. NoCA, no childhood abuse; CA, childhood abuse; BMI, body mass index

In male participants at age 19 from IMAGEN, we found the smaller FPC cluster was associated with both higher BMI (*β* = − .086, 95%CI − .128 to − .043; *p* < .001; Fig. 4a) and CA (*β* = − .568, 95%CI − .942 to − .194; *p* = .003; Fig. 4b), in a linear regression model including BMI, CA, and PRS as predictors for FPC volume (*n* = 279). We identified a significant mediation effect of the volume of FPC on the association between childhood abuse and higher BMI (indirect effect = .542, standard error [SE] = .229, 95%CI = .164–1.064), explaining 46.4% of the total effect. Longitudinally, we found a cross-lagged correlation from baseline FPC to follow-up BMI in males with a history of CA (Volume_14_ to BMI_19_: *β* = − .299, 95%CI − .599 to − .020; *p* = .042, FDR-p = .042; *n* = 62; Fig. 4c and Additional file 1: Figure S4), and this correlation was weakened in males without such a history (β_ca_–β_no-ca_ = .324, 95%CI .045 to .640; *p* = .03). Across both groups, the CA-FPC interaction at baseline was associated with weight gain (∆BMI) during the 5-year follow-up (interaction term: *β* = − .343, 95%CI − .635 to − .051; *p* = .02; Fig. 4d; polygenetic risk: *β* = .367, 95%CI .040 to .694; p = .03; the whole model: adjusted-*R*^2^ = 5.8%; *p* = .005; *n* = 278; Additional file 1: Table S9).

## Discussion

We found that volumetric shrinkage of the FPC (BA10 and BA32) quantitatively linked CA to adult overweight or obesity. As the FPC is at the apex of a cognitive and motivational control hierarchy [56], the current findings provide a neuroanatomical basis for the hypothesis that cognitive deficits after CA contribute significantly to adult obesity (i.e., the “NcC pathway”). Our observation that brain changes precede excessive weight gain may indicate that strengthening higher order cognitive control systems could decrease the risk for developing obesity in this population.

Remarkably, our findings suggest a novel top-down control pathway underlying CA-induced excessive weight gain. This fronto-polar region of the prefrontal cortex has been placed at the apex of the cognitive control hierarchy [57–59]. The lateral FPC has been related to inhibitory control, while the medial FPC has been associated with both emotion and reward processing [59]. Notably, the region we identified was located in the transition zone between the lateral FPC and medial FPC and so may participate in both processes. Thus, some studies have found, associated with excessive future weight gain a few years later, increased activation of reward regions occurring in response to palatable food [60, 61] or food cues [62, 63], while others have found that reduced volumes [64] or abnormal activity [65] in the prefrontal inhibitory control regions could predict future increases in BMI. In this study, we did not employ any cognitive or behavioral tests to enable us to determine which were the most important of these behavioral factors.

The findings on FPC provide new evidence on how higher-order cognitive and motivational control may be essential for success in weight management [10]. This may be relevant for “resisting temptation”: the frontal pole has been implicated in deciding to avoid situations requiring difficult motivational choices—the temporal discounting of reward [66]—consistent with hierarchical models of cognitive control [56]. This could correspond, for example, to better adherence to dietary strategies of avoiding exposure to appealing yet high calorie food [57]. Control of food intake could have unique features compared with controlling other behavior, as human choice strategies may have been shaped by evolution to take as much food as is encountered. This may be particularly true when dieting begins to produce weight loss, which has been associated with decreased leptin and increased ghrelin levels in blood, changes that have been associated with increased PFC activity in response to food cues [67]. Our results may partly explain why dieting for long-term weight management is so difficult [68]. Instead of repeatedly resisting opportunities to eat food encountered in the environment, higher-order cognitive control via FPC could enable the strategic avoidance of fast-food outlets and even the kitchen (and opening the refrigerator) when trying to lose weight [66]. Cognitive control in 3-year-olds predicts their weight status [69] 30 years [70] later, and our finding of reduced FPC volume associated with higher BMI may provide a neuroanatomical basis for this observation. In the extreme case of anorexia nervosa, the frontal pole is over-activated upon presentation of high-calorie stimuli compared to healthy controls [71].

Notably, the finding of a strong white-matter connection between the FPC and the eating behavior center (i.e., the hypothalamus) [72] provides a structural basis for the neurocognitive control of eating behavior. The FPC cluster we identified is located in the medial prefrontal cortex (mPFC; mainly BA24, BA25, BA32, and BA10) [8], and this network provides the origin of most of frontal projections to the hypothalamus in both rats [73] and monkeys [9]. It has long been hypothesized that the communication between the hypothalamus and the cortical regions may influence food choices, based on the observations that the reward value of food can be influenced by metabolic state [74]. Human resting-state functional connectivity has been reported between the PFC and the hypothalamus [75], and there is evidence for structural connectivity between these regions in the primate brain [9], but this has not yet been confirmed for the human brain due to the small volume of the hypothalamus and the complexity of its connectivity [76, 77]. Using 7-T dMRI data from a large sample (*n* = 156), with high spatial resolution (voxel size = 1.05 × 1.05 × 1.05 mm^3^), we found that the most polar subdivision of BA10 (i.e., R_10pp) ranked as the third most connected cortical area to the hypothalamus, especially the lateral hypothalamus among 360 cortical regions defined by the latest HCP-MMP (multi-modal parcellation) atlas. This finding may generate a testable hypothesis that the strength of this connection may be associated with the excessive weight gain, especially after the exposure to childhood abuse. We could not test this association using the HCP sample, since the childhood trauma was not assessed in this cohort.

Our findings suggest that larger FPC volume may be protective against higher BMI associated with CA. The smaller the FPC volume, the stronger was the abuse-brain association across different data collection sites, and the increased abuse-brain association was in turn correlated with the stronger abuse-BMI association. We also demonstrated that a smaller FPC volume at baseline significantly contributed to the prediction of weight gain at follow-up. Our sexually dimorphic results provide further support that the smaller the FPC volume, the greater were the effect sizes of these associations. The FPC volume was smaller in male than in female participants, which is consistent with a 2014 meta-analysis of sex difference at a regional level [19]. We had similar statistical power for both female (*n* = 325) and male (*n* = 314) participants in IMAGEN, but we could identify a significant FPC cluster associated with CA in males only, indicating the effect size of the abuse-brain association was smaller in the females from the IMAGEN sample. The effect size of the abuse-BMI association was also smaller in female than in male participants at a trend level (*p* = .08) in this sample. Both the abuse-FPC and abuse-BMI associations became significant in the much larger and also older UKB sample of female participants (*n* = 2396). The identified sex differences are also supported by both animal models for childhood trauma [78] and human epidemiological studies on the trauma-obesity association [79].

These findings of sex difference may suggest that sex hormones such as testosterone might be involved in the excessive weight gain in adolescent males after the exposure childhood trauma. Low testosterone levels have been implicated in metabolic dysfunction, especially associated with increased central adiposity and reduced lean mass in males, while weight loss has also been linked to increased testosterone levels [80]. Childhood trauma has been associated with negative testosterone-cortisol coupling in adolescent females, but positive testosterone-cortisol coupling in adolescent males [81], since childhood trauma can alter the coupling between hypothalamic pituitary adrenal axis and hypothalamus-pituitary-gonadal [82].

The present findings may be important clinically. Consistent with previous reports [14, 83], the CA prevalence was as high as 1 in 4 young male participants in our sample, and the risk of being overweight or obese at age 19 was 2.17-fold higher than those without exposure to CA (*n* = 314, OR = 2.168, 95%CI 1.244 to 3.777; *p* = .006). Non-invasive brain stimulation therapies [84], such as transcranial magnetic stimulation and transcranial direct current stimulation, are already applied for the treatment of obesity and eating disorders. To date, most studies have targeted the dorsolateral PFC, but therapeutic effects have been inconclusive [84]. Our findings therefore identify the FPC as a novel brain target for the treatment of obesity.

### Limitations

The present study had several limitations. First, because this is an observational study, any suggestion of causal associations must be considered with caution. Second, given the complex role of the frontopolar cortex, future neuroimaging experiments are required to examine effects on down-stream networks. Third, detailed cognitive or behavioral assessments will be useful in future studies to elucidate the contributions of different control functions to excessive weight gain after childhood abuse.

## Conclusions

We have identified volumetric reduction in FPC as a key neuroanatomical link between childhood abuse and adult obesity. This finding highlights the importance of higher-order cognitive control in weight management. Development of cognitive intervention strategies to compensate for the possible resultant functional deficiencies is warranted.

## Supplementary information

**Additional file 1. **Supplementary methods, tables, figures and appendix. **Table S1.** Demographic characteristics of the participants from the UK Biobank. **Table S2.** Relationship between childhood abuse and BMI, in male participants in the IMAGEN study. **Table S3.** Significant clusters after permutation-based TFCE correction, in the male participants from IMAGEN study. **Table S4.** Relationship between childhood abuse and FPC volume, in male participants in the IMAGEN study. **Table S5.** Effect size of environmental or genetic risk on BMI, and the significance of difference between the IMAGEN and UK Biobank samples. **Table S6.** Significant clusters after permutation-based TFCE correction, in the male participants from the UK Biobank. **Table S7.** Significant clusters after permutation-based TFCE correction, in the female participants from the UK Biobank dataset. **Table S9.** Predictability of baseline information to BMI change between baseline and follow-up. **Figure S1.** Hypothalamus defined by the CIT168 atlas. **Figure S2.** Association between abuse-brain association and abuse-BMI association in females. **Figure S3.** Structural connectivity of the lateral and the medial hypothalamus. **Figure S4**. Cross-lagged path analyses between frontopolar volume and BMI in IMAGEN.

**Additional file 2: Table S8.** White matter connections between the hypothalamus and the cortical and subcortical areas tracked using the HCP-7T dMRI data with a high spatial resolution.

## Data Availability

The IMAGEN data are available by application to the consortium coordinator Dr. Schumann (http://imagen-europe.com) after evaluation according to an established procedure. UK Biobank is an open Resource and is available to researchers by registering and applying to access the Resource via the Resource Access Management System (http://www.ukbiobank.ac.uk/). This research has been conducted using the UK Biobank Resource under application 19542. The 7-T DTI data were provided by the Human Connectome Project, WU-Minn Consortium (Principal Investigators: David Van Essen and Kamil Ugurbil; https://www.humanconnectome.org/).
