# Supplementary material for: Association between childhood trauma and risk for obesity: a putative neurocognitive developmental pathway
Source: BMC Med. 2020 Oct 15;18:278. doi: 10.1186/s12916-020-01743-2 (PMC7559717; doi:10.1186/s12916-020-01743-2)
Supplement: Supplementary file 1 — Additional file 1. Supplementary methods, tables, figures and appendix. Table S1. Demographic characteristics of the participants from the UK Biobank. Table S2. Relationship between childhood abuse and BMI, in male participants in the IMAGEN study. Table S3. Significant clusters after permutation-based TFCE correction, in the male participants from IMAGEN study. Table S4. Relationship between childhood abuse and FPC volume, in male participants in the IMAGEN study. Table S5. Effect size of environmental or genetic risk on BMI, and the significance of difference between the IMAGEN and UK Biobank samples. Table S6. Significant clusters after permutation-based TFCE correction, in the male participants from the UK Biobank. Table S7. Significant clusters after permutation-based TFCE correction, in the female participants from the UK Biobank dataset. Table S9. Predictability of baseline information to BMI change between baseline and follow-up. Figure S1. Hypothalamus defined by the CIT168 atlas. Figure S2. Association between abuse-brain association and abuse-BMI association in females. Figure S3. Structural connectivity of the lateral and the medial hypothalamus. Figure S4. Cross-lagged path analyses between frontopolar volume and BMI in IMAGEN. [file 12916_2020_1743_MOESM1_ESM.docx]

Supplementary Information

**Association Between Childhood Trauma and Risk for Obesity: A Putative Neurocognitive Developmental Pathway**

Qiang Luo, et al.

**Table of Content**

[Supplementary methods 2](#_Toc46568817)

[Method S1. Discovery sample from the IMAGEN study 2](#_Toc46568818)

[Method S2. Validation Sample from the UK Biobank 7](#_Toc46568819)

[Method S3. White matter fiber tracking for hypothalamus using 7T HCP dMRI data 9](#_Toc46568820)

[Supplementary tables 12](#_Toc46568821)

[Table S1. Demographic characteristics of the participants from the UK Biobank 12](#_Toc46568822)

[Table S2. Relationship between childhood abuse and BMI, in male participants in the IMAGEN study, adjusting for potential confounders/covariates. 13](#_Toc46568823)

[Table S3. Significant clusters after permutation-based TFCE correction, in the male participants from IMAGEN study. 14](#_Toc46568824)

[Table S4. Relationship between childhood abuse and FPC volume, in male participants in the IMAGEN study, adjusting for potential confounders/covariates. 15](#_Toc46568825)

[Table S5. Effect size of environmental or genetic risk on BMI, and the significance of difference between the IMAGEN and UK Biobank samples. 16](#_Toc46568826)

[Table S6. Significant clusters after permutation-based TFCE correction, in the male participants from the UK Biobank 17](#_Toc46568827)

[Table S7. Significant clusters after permutation-based TFCE correction, in the female participants from the UK Biobank dataset 18](#_Toc46568828)

[Table S8. White matter connections between the hypothalamus and the cortical and subcortical areas tracked using the HCP-7T dMRI data with a high spatial resolution. 19](#_Toc46568829)

[Table S9. Predictability of baseline information to BMI change between baseline and follow-up. 19](#_Toc46568830)

[Supplementary figures 20](#_Toc46568831)

[Figure S1. Hypothalamus defined by the CIT168 atlas. 20](#_Toc46568832)

[Figure S2. Association between abuse-brain association and abuse-BMI association in females. 22](#_Toc46568833)

[Figure S3. Structural connectivity of the lateral and the medial hypothalamus. 23](#_Toc46568834)

[Figure S4. Cross-lagged path analyses between frontopolar volume and BMI in IMAGEN. 24](#_Toc46568835)

[Appendix 25](#_Toc46568836)

# Supplementary methods

## Method S1. Discovery sample from the IMAGEN study

The participants in this study were recruited by the IMAGEN study, a European multi-center longitudinal research project on healthy youth (<http://imagen-europe.com/>). All participants' parents gave written informed consent after information on the research procedures, and adolescents gave their assent after written information. Childhood maltreatment was assessed at follow-up (age 19) for 1183 participants by June 2016, of which 1159 had no missing data. The BMI information was available for 1042 participants at both data collection waves. After excluding 87 underweight participants (BMI <18.5kg/m2) at the follow-up, 955 participants remained. We had the structural neuroimages from 949 participants whose images passed the quality check of sample homogeneity during the image preprocessing with VBM8 at both data collection waves. Finally, we had 639 adolescents (of whom 325 were female adolescents) with the qualified data of the childhood trauma, BMI, and the structural neuroimaging at both data collection waves. Of these, 557 adolescents (278 females) had genetic information, 542 (278 females) had lifetime drug use information, and 588 (303 females) had depressive scores.

**Environmental and behavioral indices**

**Childhood trauma** The 28-item Childhood Trauma Questionnaire (CTQ,[29]) was used to assess childhood trauma when the participants were at age 19. The CTQ is a retrospective, self-report measure, including five subscales: emotional abuse (EA), physical abuse (PA), sexual abuse (SA), emotional neglect (EN), and physical neglect (PN). All items were scored on a 5-point ordinal scale to express the frequency of the maltreatment with 1 being “never true” to 5 being “very often true”. The five subscale scores were then calculated by adding up the corresponding items, and scored from 5 to 25. Since the participants in the IMAGEN study were healthy subjects, we used the lowest cut-off as 8 for EA, 7 for PA, 5 for SA, 9 for EN, and 7 for PN[1]. If any one of the EA, PA, SA happened, the abuse scored one; otherwise, scored zero. Similarly, the neglect score was calculated to be one, if any of the EN or PN happened.

**Additional environmental or behavioral indices** Drug use behavior was measured using European School Survey Project on Alcohol and Drugs (ESPAD). We focused on the items of lifetime drug use at the follow-up. Participants who took either type of the drugs (marijuana, inhalants, tranquilisers, amphetamines, LSD, magic mushrooms, crack, cocaine, heroin, narcotics, MDMA, ketamine, GHB, anabolic steroids) were scored one; otherwise, zero. Depressive score was measured with Adolescent Depression Rating Scale (ADRS) at the follow-up. Family socioeconomic status was surveyed by the Development and Well-Being Assessment [30], the socioeconomic/housing score from the family stresses subsection at baseline. The Life Event Questionnaire[31] was used to record the occurrence of stressful events, both lifetime and during the previous 12 months. We focus on the recent stressful events, using the number of stressful life events that had occurred during the previous 12 months. Birth weight in grams was collected by the Pregnancy and Birth Questionnaire [31] at baseline.

**Structural neuroimaging data**

**Structural image acquisition** MRI was performed on 3 Tesla scanners from six study sites. Details of the MRI acquisition protocols have been described previously, including an extensive period of standardization across data collection centers[22]. In this study, we used the T1-weighted images. These high-resolution anatomical MRIs were obtained using a three-dimensional magnetization prepared rapid acquisition gradient-echo sequence (MPRAGE) based on the ADNI protocol <http://adni.loni.usc.edu/methods/documents/mri-protocols/>) with the following acquisition parameters: repetition time = 2300ms, echo time = 2.8ms, flip angle = 8°; 240×256×160 matrix, 1.1×1.1×1.1 mm voxel size).

**Voxel-based morphometry** All data were preprocessed in SPM8 (http://www.fil.ion.ucl.ac.uk/spm/) using the VBM8 toolbox with default settings, including the usage of high-dimensional spatial normalization with an already integrated Dartel template in MNI space and segmented into different tissue types by the new segmentation algorithm in the VBM[33]. All images were subjected to nonlinear modulations and corrected for each individual head size. Images were then smoothed with an 8 mm full-width at half-maximum Gaussian kernel with the resulting voxel size 1.5 mm3.The TIV was estimated by the summation of the grey matter, white matter, and CSF volumes in native space. The automated anatomical labeling (AAL) atlas was employed to exclude the voxels outside the grey matter.

**Genome-wide genotype data**

Whole-blood samples were collected in 2,087 IMAGEN participants. The genotyping and quality controlling procedures have been described in a previous study [34]. DNA purification and genotyping were performed by the Centre National de Génotypage in Paris. A total of 705 and 1,382 individuals were genotyped with the Illumina (Little Chesterford, UK) Human610-Quad Beadchip (582,982 SNPs) and Illumina Human660-Quad Beadchip (557,124 SNPs), both of which were based on the same Illumina HumanHap550 Genotyping BeadChip with varied additional probes, and therefore they have most of their SNPs (551,141 SNPs) in common, with the same rsID and location based on assembly hg18. For each genotyping platform the following quality control was performed separately. Single-nucleotide polymorphisms (SNPs) with call rates < 95%, minor allele frequency < 5%, deviation from the Hardy–Weinberg equilibrium (p < 1×10^-3^) and nonautosomal SNPs were excluded from the analyses. Individuals with excessive missing genotypes (failure rate > 5%) were also excluded. Population homogeneity was examined with the Structure software using HapMap populations as reference groups. Individuals with divergent ancestry (from Utah residents with ancestry from northern and western Europe) were excluded. Identity-by-state clustering and multi-dimensional scaling were used to estimate cryptic relatedness for each pair of individuals using the PLINK software and closely related individuals were eliminated from the subsequent analysis. We applied principal component analysis to remove remaining outliers, defined as individuals located at more than four s.d. of the mean principal component analysis scores on one of the first 20 dimensions. Before merging, strand flipping was conducted to make sure that the same strand was used to recode the same genotypes from both platforms. Genotypes from both platforms were then combined through software PLINK where platform-specific SNPs were removed. After the quality control measures, we obtained a total of 466, 114 SNPs in 1834 individuals, which then used for the subsequent polygenic risk scoring without imputation.

Polygenic risk scores (PRSs) for higher BMI (PRS_BMI_) were calculated using the PRSice software (<http://prsice.info/>)[35]. To generate PRS_BMI_ in IMAGEN, we used publicly available data from the GIANT Consortium, which included 2,554,637 SNPs and up to 339,224 individuals of European ancestry[36]. These two cohorts shared 462,170 SNPs. The SNPs with the smallest *p*-value for each linkage disequilibrium block (excluding SNPs with an r^2^<.1 in 250 kb windows) were retained after clumping; and the PRSs were calculated at the *p*-value threshold of .05.

**Temporal precedence**

We built a linear regression model to predict the weight gain (BMI_14_ -BMI_19_) during 5-year follow-up using the baseline Volume_14_ of the identified neural correlates. Both the main effects and the interaction terms were considered in this model. The covariates used in these models included PRS_BMI_, family’s socioeconomic status, handedness, scanning sites, and baseline TIV.

## Method S2. Validation Sample from the UK Biobank

We used a large adult database (from the UK Biobank) to test whether the relationship among childhood trauma, higher BMI, and brain structural alteration of the IMAGEN data are robust and could be validated in and extended to an older aged population. We focused on a subsample of participants with structural images, released on February 2017 (n = 9,888). The inclusion criteria included: 1) participants with age from 45 to 65-year-old, 2) with BMI information, 3) with the childhood maltreatment assessments. The exclusion criterion was that participants with a BMI <18.5 kg/m2, in line with the IMAGEN study. After quality control for neuroimaging data and behavioral assessments, 4121 participants (2396 females) were included in the current study (*Table S1*). Of these, 2933 participants (1697 females) had genetic information and entered the analyses with genetic information involved.

**Environmental and behavioral indices**

**Anthropometric indices** Participants’ BMI were collected during the imaging visit.

**Childhood trauma** Childhood traumatic events were measured with the following items: physically abused by family member as a child (Field ID: 20488); felt hated by family members as a child (Field ID: 20487); and sexually molested as a child (Field ID: 20490). If any type of abuse reported, the abuse scored one; otherwise, zero.

**Structural imaging data**

Details of the structural MRI acquisition protocols and image preprocessing procedures have been described previously [33]**.** The structural MRI images were released on February 2017, collected with a single scanner dedicated to UKB imaging in Cheadle Manchester. The scanner is a standard Siemens Skyra 3T running VD13A SP4 (as of October 2015), with a standard Siemens 32-channel RF receive head coil. The imaging protocol is as follows: Resolution: 1×1×1mm Field-of-view: 208×256×256 matrix Duration: 5 minutes 3D MPRAGE, sagittal, in-plane acceleration iPAT=2, prescan-normalise. For more details we refer to the official document for neuroimaging of the UKB (http://biobank.ctsu.ox.ac.uk /crystal/docs/brain_mri.pdf). To be consistent, we followed exactly the same pre-processing procedure as the IMAGEN study using the VBM8. Then voxel-wise regression models were used to estimate the association of GMV in the brain with either childhood abuse, PRS_BMI_ or BMI, following the same approaches conducted in the IMAGEN. In these models, we included age and total intracranial volume as covariates. Multiple comparisons were adjusted using the voxel-wise non-parametric permutation test (5000 permutations, no acceleration method) with TFCE (p < 0.05, 1-tailed test).

**Genome-wide genotype data**

The genotyped data we used in this study was released on July 2017. Genotype data are available for all 500,000 participants in the UKB cohort. Genotyping was performed using the Affymetrix UK BiLEVE Axiom array on an initial 50,000 participants; the remaining 450,000 participants were genotyped using the Affymetrix UK Biobank Axiom® array. The two arrays are extremely similar (with over 95% common content). Samples used in this study were estimated to have recent British ancestry and have no more than 10 putative third-degree relatives in the kinship table using the sample quality control information provided centrally by UKB (using the variables of *white.British.ancestry.subset* and *excess.relatives* in the file *ukb_sqc_v2.txt*). Following the quality control procedures described previously [37], we obtained a total of 602, 821 SNPs without imputation in 337,199 individuals.

## Method S3. White matter fiber tracking for hypothalamus using 7T HCP dMRI data

The scanning protocol and the preprocessing pipeline are both detailed in the Reference Manual for the HCP-S1200-Release, which can be found in the following link (<https://humanconnectome.org/storage/app/media/documentation/s1200/HCP_S1200_Release_Reference_Manual.pdf>). We also briefly introduced below.

The data were collected by HCP using a Siemens Magnetom 7T MR scanner with Nova32 32-channel Siemens receive head coil at the Center for Magnetic Resonance (CMRR) at University of Minnesota in Minneapolis, MN[38]. The Spin-echo EPI sequence was used for dMRI with a multi-shell diffusion scheme (TR = 7000ms, TE=71.2ms, flip angle = 90 deg, refocusing flip angle = 180 deg, FOV = 210×210, matrix = 200×200, slice thickness = 1.05mm, 132 slices, 1.05 mm isotropic voxels, multiband factor = 2, image acceleration factor = 3, Echo spacing = 0.82ms, b-values = 1000 and 2000 s/mm^2^). The dMRI session included 4 runs with 9 minutes and 50 seconds each for 2 different gradient tables and 2 phase encoding polarities (i.e. anterior-to-posterior and posterior-to-anterior). Each gradient table included approximately 65 diffusion weighting directions plus 6 b=0 acquisitions. The diffusion directions were given by a toolbox available from Emmanuel Caruyer that returns uniformly distributed directions in multiple q-space shells (http://www.emmanuelcaruyer.com/q-space-sampling.php).

The preprocessing pipeline included the following steps [39-41]: 1) normalizes intensity of b0 images; 2) removes EPI distortions by ‘TOPUP’ algorithm and both eddy-current-induced distortions and subject motion by ‘EDDY’ algorithm (v5.0.10); 3) corrects for gradient-nonlinearities; 4) registers the diffusion data with the T1w data with FLIRT BBR+bbregister; transforms it into 1.25mm structural space; and masks the data with the final brain mask based on FreeSurfer segmentation. HCP pipelines v3.19.0 is also available on Github (https://github.com/Washington-University/HCPpipelines).

The diffusion data were reconstructed using generalized q-sampling imaging[42] with a diffusion sampling length ratio of 1.25. A deterministic fiber tracking algorithm[43] was used. To trace the fiber tracts from hypothalamus, the seeding region was placed at the hypothalamus that defined by the CIT168 (California Institute of Technology) atlas, which is a high-resolution probabilistic *in vivo* atlas of human subcortical brain nuclei constructed using neuroimaging data from 168 participants (eFigure 1)[44]. A total of 10,000 streamlines were reconstructed for each subject with an anisotropy threshold 0.02 (qa), an angular threshold 60 deg, and a step size 0.5 mm. Streamlines with the length shorter than 30 or longer than 300 mm were discarded. The whole-brain connectome for hypothalamus was generated, using the cortical areas defined by the HCP-MMP (multi-modal parcellation) atlas (which has been constructed using the high-resolution HCP neuroimaging data[45]), 7 subcortical areas defined by FreeSurfer (thalamus proper, caudate, putamen, pallidum, hippocampus, amygdala, and nucleus accumbens) and the hypothalamus was defined by the CIT168 atlas. In this study, we further divided the hypothalamus into eight subregions (four for each hemisphere) based on the coordinates of the center of mass (left: [-5, -3,-10], right: [4, -3, -10]). The anterior medial (AM) part is defined as the part of the hypothalamus in front of the coronal plane (Y ≥ -3) and medial between two sagittal planes (X= -5 ~ 4), while the anterior lateral (AL) is lateral to the sagittal plane in each hemisphere (left: X< -5; right X > 4). The posterior part is defined for regions behind the coronal plane (Y < -3), in which the posterior medial (PM) part is the region between two sagittal planes: X=-5 and X=4, while posterior lateral (PL) is defined for regions lateral to the sagittal planes: X < -5 or X > 4 for left and right hemisphere. Left and right subdivisions are defined by the middle sagittal plane (X=0).

The connectivity matrix was calculated by using the count of the connecting tracks that end in paired parcels (mean fa connectivity was also calculated). The connectivity matrix was conducted using DSI Studio (<http://dsi-studio.labsolver.org>). Among 178 participants in this database, 22 of them were excluded from further analysis, since they had at least one subdivision of HTH without any brain connectivity can be tract. The hypothalamic connectivity profile (374 brain regions × 156 subjects) was extracted directly from the whole-brain connectivity matrix of each subject. To assess the significance of the connectivity tracked, we calculated a ratio of connectivity

and a ratio of size

Combining one-sample sign test with false discovery rate, we assessed whether the median of ratio of connectivity was greater than the ratio of size, because if the connectivity we found was randomly distributed among all brain areas, the distribution of the fiber numbers estimated was expected to be defined merely according to the sizes of these brain areas. We also compared the cortical connections between the lateral and the medial hypothalamus by combining two-sample sign test of median with false discovery rate.

# Supplementary tables

## Table S1. Demographic characteristics of the participants from the UK Biobank

| **Participants** ^a^ | **Older males** | | **Older females** | |
| --- | --- | --- | --- | --- |
|  | **No exposure** | **Exposure** | **No exposure** | **Exposure** |
| **Childhood Abuse** | 1126 | 599 | 1526 | 870 |
| **Body Mass Index** | 27.03±3.90 | 27.76±4.48 | 26.10±4.70 | 27.13±5.17 |
| **PRS_BMI_** | -7.022×10^-3^  (1.237×10^-3^) | -7.113×10^-3^  (1.235×10^-3^) | -7.056×10^-3^  (1.322×10^-3^) | -7.051×10^-3^  (1.256×10^-3^) |

^a^ Numbers of subjects with a particular characteristic are listed as integers, and quantitative measurements are presented as mean values ± standard deviations.

## Table S2. Relationship between childhood abuse and BMI, in male participants in the IMAGEN study, adjusting for potential confounders/covariates.

| **Relationship between childhood abuse and Follow-up BMI in males** ^a^ | | |
| --- | --- | --- |
| **Confounder/covariate ^a^** | **t** | **p** |
| PRS_BMI_ | 2.097 | 0.037 |
| Family SES | 2.764 | 0.006 |
| Current life stress | 2.711 | 0.007 |
| Birth weight | 2.332 | 0.021 |
| Illegal drug use | 2.029 | 0.043 |
| Depressive score | 2.377 | 0.018 |

^a^ adjusted coefficients and significance are presented. Adjustment of the effect of PRS_BMI_ (n=279),family socioeconomic status (n = 313), current life stress (n = 305), birth weight (n = 250), illegal drug use (n = 264), or depressive score (n = 285) was conducted separately.

## Table S3. Significant clusters after permutation-based TFCE correction, in the male participants from IMAGEN study.

| **Region** | **Cluster size (voxels)** | **Peak t value** | **MNI coordinates (Peak)** | | | |
| --- | --- | --- | --- | --- | --- | --- |
|  |  |  | **x** | **y** | **z** | |
| **Regions significantly associated with body mass index (n = 314)** | | | | | | |
| *Frontal_Sup_Orb_R* | 191920 | -5.996 | 9 | 69 | -6 | |
| **Regions significantly associated with childhood abuse experience (n = 314)** | | | | | | |
| *Frontal_Med_Orb_L* | 8712 | -3.874 | -7.5 | 69 | | -6 |
| *Temporal_Pole_Sup_L* | 762 | -4.606 | -27 | 9 | | -25.5 |
| **Regions significantly associated with polygenic risk score for obesity at the threshold of p < 0.05 (PRS_BMI_ P_T_ < 0.05) (n = 279)** | | | | | | |
| *Frontal_Sup_Medial_L* | 3525 | -4.337 | -3 | 49.5 | | 16.5 |
| *Heschl_L* | 1862 | -4.863 | -57 | -10.5 | | 9 |
| *Lingual_R* | 879 | -4.426 | 12 | -82.5 | | -10.5 |
| *Lingual_L* | 338 | -3.936 | -21 | -46.5 | | -1.5 |

^a^ p < 0.05 after correction for multiple comparisons, using the permutation-based threshold-free cluster enhancement (TFCE) approach.

## Table S4. Relationship between childhood abuse and FPC volume, in male participants in the IMAGEN study, adjusting for potential confounders/covariates.

| **Relationship between childhood abuse and Follow-up FPC volume in males** ^a^ | | |
| --- | --- | --- |
| **Confounder/covariate ^a^** | ***β* (95%CI)** | **p** |
| PRS_BMI_ | -0.675 (-1.055,-0.295) | 0.0005 |
| Family SES | -0.723 (-1.082,-0.364) | 9.20×10^-5^ |
| Current life stress | -0.730 (-1.094,-0.365) | 1.02×10^-4^ |
| Birth weight | -0.712 (-1.118,-0.306) | 0.0005 |
| Illegal drug use | -0.732 (-1.135,-0.328) | 4.21×10^-4^ |
| Depressive score | -0.728 (-1.112,-0.344) | 2.44×10^-4^ |

^a^ adjusted coefficients, their 95% confidence intervals(CI) and significance are presented. Adjustment of the effect of PRS_BMI_ (n=279), family socioeconomic status (n = 313), current life stress (n = 305), birth weight (n = 250), illegal drug use (n = 264), or depressive score (n = 285) was conducted separately.

## Table S5. Effect size of environmental or genetic risk on BMI, and the significance of difference between the IMAGEN and UK Biobank samples.

| **Effect size**  ***(Cohen’s d)*** | | **Childhood abuse** | | | | **Genetic risk** | | | |
| --- | --- | --- | --- | --- | --- | --- | --- | --- | --- |
|  |  | **Male** | **Female** | ***Sex Difference*** | | **Male** | **Female** | ***Sex Difference*** | |
|  |  |  |  | ***z*** | ***p*** |  |  | ***z*** | ***p*** |
| **Imagen** | | 0.316 | 0.116 | -1.755 | 0.079 | 0.650 | 0.367 | -2.082 | 0.037 |
| **UK Biobank** | | 0.166 | 0.206 | 0.835 | 0.404 | 0.411 | 0.377 | -0.643 | 0.521 |
| ***Sample***  ***Difference*** | ***z*** | -1.702 | 1.063 | / | / | -2.470 | 0.103 | / | / |
|  | ***P*** | 0.089 | 0.287 | / | / | 0.014 | 0.918 | / | / |

## Table S6. Significant clusters after permutation-based TFCE correction, in the male participants from the UK Biobank

| **Region** | **Cluster size (voxels)** | **Peak t value** | **MNI coordinates (Peak)** | | | |
| --- | --- | --- | --- | --- | --- | --- |
|  |  |  | **x** | **y** | **z** | |
| **Regions significantly associated with body mass index (n = 1725)** | | | | | | |
| *Frontal_Sup_R* | 265188 | -14.381 | -6 | -16.5 | 1.5 | |
| **Regions significantly associated with childhood abuse experience (n = 1725)** | | | | | | |
| *Parietal_Inf_L* | 1041 | -5.071 | -45 | -21 | | 37.5 |
| *Postcentral_R* | 795 | -4.560 | 48 | -16.5 | | 37.5 |
| *Lingual_R* | 690 | -4.670 | 9 | -34.5 | | -4.5 |
| *SupraMarginal_L* | 601 | -4.024 | -55.5 | -28.5 | | 19.5 |
| *Lingual_L* | 587 | -4.532 | -10.5 | -39 | | -3 |
| *Parietal_Inf_R* | 374 | -4.937 | 39 | -39 | | 55.5 |
| *Frontal_Sup_R* | 246 | -3.877 | 25.5 | 57 | | 10.5 |
| **Regions significantly associated with polygenic risk score for obesity at the threshold of p < 0.05 (PRS_BMI_) (n = 1236)** | | | | | | |
| *Frontal_Med_Orb_R* | 9886 | -5.142 | 12 | 63 | | -1.5 |
| *Frontal_Inf_Orb_R* | 1939 | -4.110 | 34.5 | 21 | | -22.5 |
| *Temporal_Inf_R* | 802 | -3.581 | 46.5 | -39 | | -18 |
| *Temperal_Mid_R* | 635 | -4.636 | 73.5 | -37.5 | | -10.5 |
| *Frontal_Sup_Orb_L* | 264 | -3.545 | -19.5 | 66 | | -6 |
| *Precentral_R* | 299 | -3.410 | 49.5 | -7.5 | | 37.5 |

^a^ p < 0.05 after correction for multiple comparisons, using the permutation-based threshold-free cluster enhancement (TFCE) approach.

## Table S7. Significant clusters after permutation-based TFCE correction, in the female participants from the UK Biobank dataset

| **Region** | **Cluster size (voxels)** | **Peak t value** | **MNI coordinates (Peak)** | | | |
| --- | --- | --- | --- | --- | --- | --- |
|  |  |  | **x** | **y** | **z** | |
| **Regions significantly associated with body mass index (n = 2396)** | | | | | | |
| *Rectus_R* | 286859 | -17.484 | 4.5 | 48 | -24 | |
| **Regions significantly associated with childhood abuse experience (n = 2396)** | | | | | | |
| *Frontal_Med_Orb_R* | 10372 | -4.034 | 4.5 | 63 | | -15 |
| *Insula_L* | 5396 | -4.423 | -45 | 3 | | 0 |
| *Temporal_Mid_L* | 478 | -3.920 | -52.5 | -10.5 | | -16.5 |
| *Temporal_Sup_R* | 451 | -3.655 | 52.5 | -39 | | 18 |
| *Temporal_Mid_R* | 379 | -3.277 | 54 | -36 | | 3 |
| *Temporal_Sup_R* | 451 | -3.655 | 52.5 | -39 | | 18 |
| **Regions significantly associated with polygenic risk score for obesity at the threshold of p < 0.05 (PRS_BMI_) (n = 1697)** | | | | | | |
| *Temporal_Sup_R* | 44442 | -5.316 | 64.5 | -10.5 | | 9 |
| *Temporal_Inf_R* | 1944 | -4.944 | -54 | -13.5 | | -33 |
| *SupraMarginal_L* | 1907 | -4.686 | -63 | -24 | | 13.5 |

^a^ p < 0.05 after correction for multiple comparisons, using the permutation-based threshold-free cluster enhancement (TFCE) approach.

## Table S9. Predictability of baseline information to BMI change between baseline and follow-up.

|  | ∆**BMI ~ childhood abuse + frontopolar volume +**  **childhood abuse*frontopolar volume + genetic risk + family SES + covs^a^** | | | |
| --- | --- | --- | --- | --- |
| **Whole Model** | **adjusted R^2^** | **F/β** | **95% CI** | **p** |
|  | 0.058 | 2.432 | / | 0.005 |
| **Variables** | **childhood abuse** | 0.228 | (-0.542,0.999) | 0.560 |
|  | **frontopolar volume** | -0.043 | (-0.178,0.093) | 0.537 |
|  | **Childhood abuse**  ***frontopolar volume** | -0.343 | (-0.635,-0.051) | 0.022 |
|  | **genetic risk** | 0.367 | (0.040,0.694) | 0.028 |
|  | **Family SES** | -0.280 | (-0.626,0.066) | 0.112 |

^a^ covs in this model included handedness, scanning sites, and baseline total intracranial volume.

# Supplementary figures

## Figure S1. Hypothalamus defined by the CIT168 atlas.

**
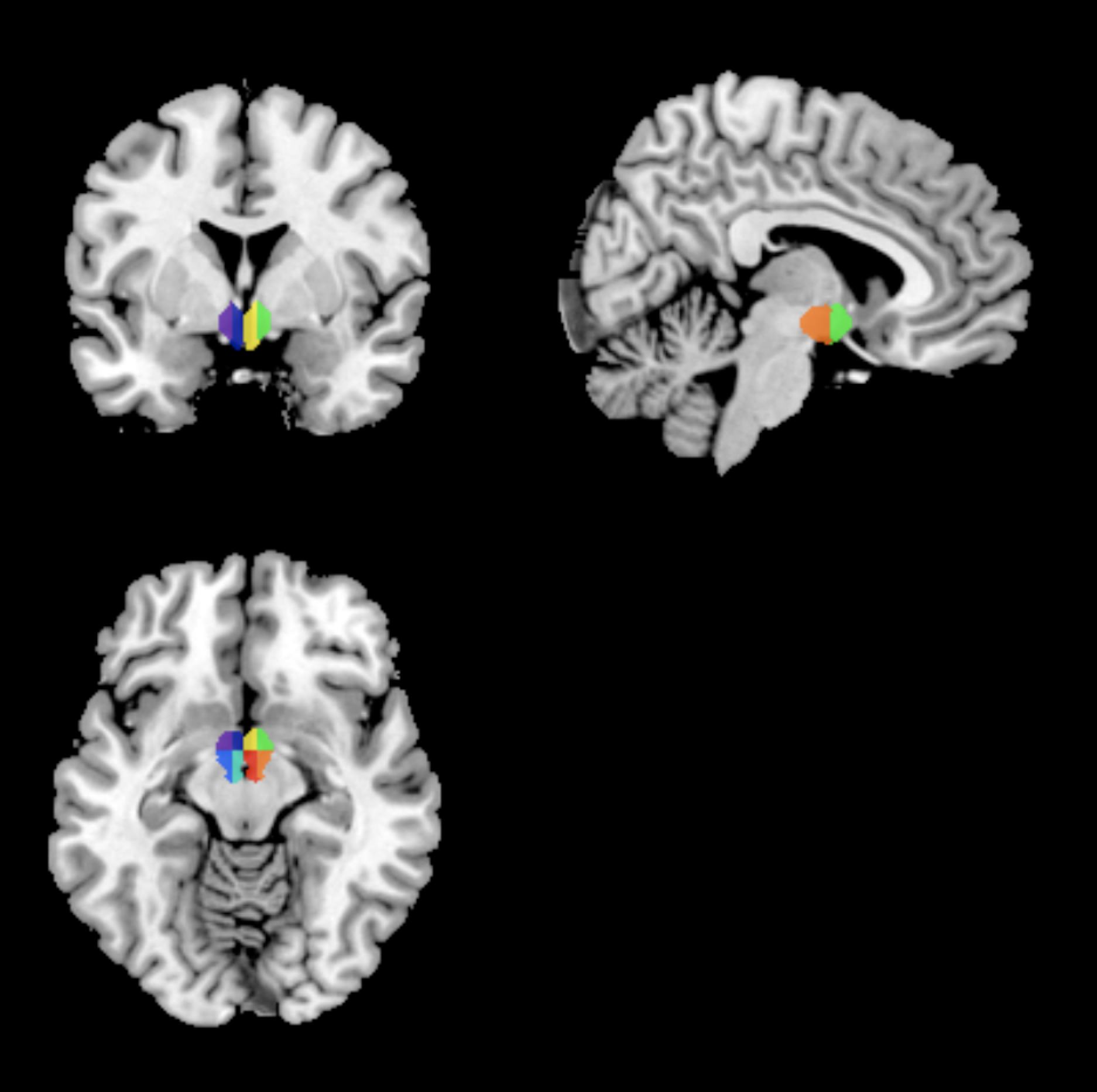
**

Sizes of the subdivisions of hypothalamus:

| Subdivision | Color | Number of Voxels |
| --- | --- | --- |
| 'l_AL' | Purple | 226 |
| 'l_AM' | Dark blue | 220 |
| 'l_PL' | Cyan | 320 |
| 'l_PM' | Light blue | 297 |
| 'r_AL' | Green | 227 |
| 'r_AM' | Yellow | 297 |
| 'r_PL' | Orange | 271 |
| 'r_PM' | Red | 342 |

l-left, r-right, A-anterior, P-posterior, L-lateral, M-medial

In this study, we divided the hypothalamus (HTH) into four parts per hemisphere, based on the center-of-mass coordinate (left: [-5, -3,-10], right: [4, -3, -10]) of the predefined HTH mask in the CIT168 reinforcement atlas. The anterior medial (AM) subdivision was defined as the part of the HTH in front of the coronal plane (Y ≥ -3) and between two sagittal planes (X= -5 ~ 4), while the anterior lateral (AL) subdivision was lateral to the sagittal plane in each hemisphere (left: X< -5; right X > 4). The posterior part was defined for the regions behind the coronal plane (Y < -3), in which the posterior medial (PM) subdivision was the region between two sagittal planes: X=-5 and X=4, while posterior lateral (PL) subdivision was defined for regions lateral to the sagittal planes: X < -5 or X > 4 for left and right hemisphere. Left and right subdivisions were defined by the middle sagittal plane (X=0).

## Figure S2. Association between abuse-brain association and abuse-BMI association in females.

**
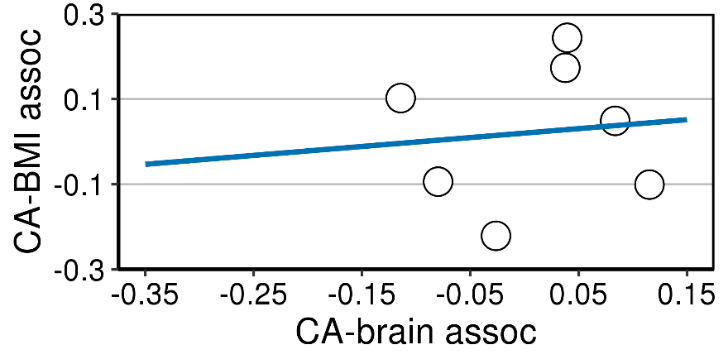
**

The partial correlation coefficient between childhood abuse and BMI was associated with the partial correlation coefficient between childhood abuse and the FPC volume in female subjects across the 6 data collection sites in the IMAGEN study and the UK Biobank sample.

## Figure S3. Structural connectivity of the lateral and the medial hypothalamus.


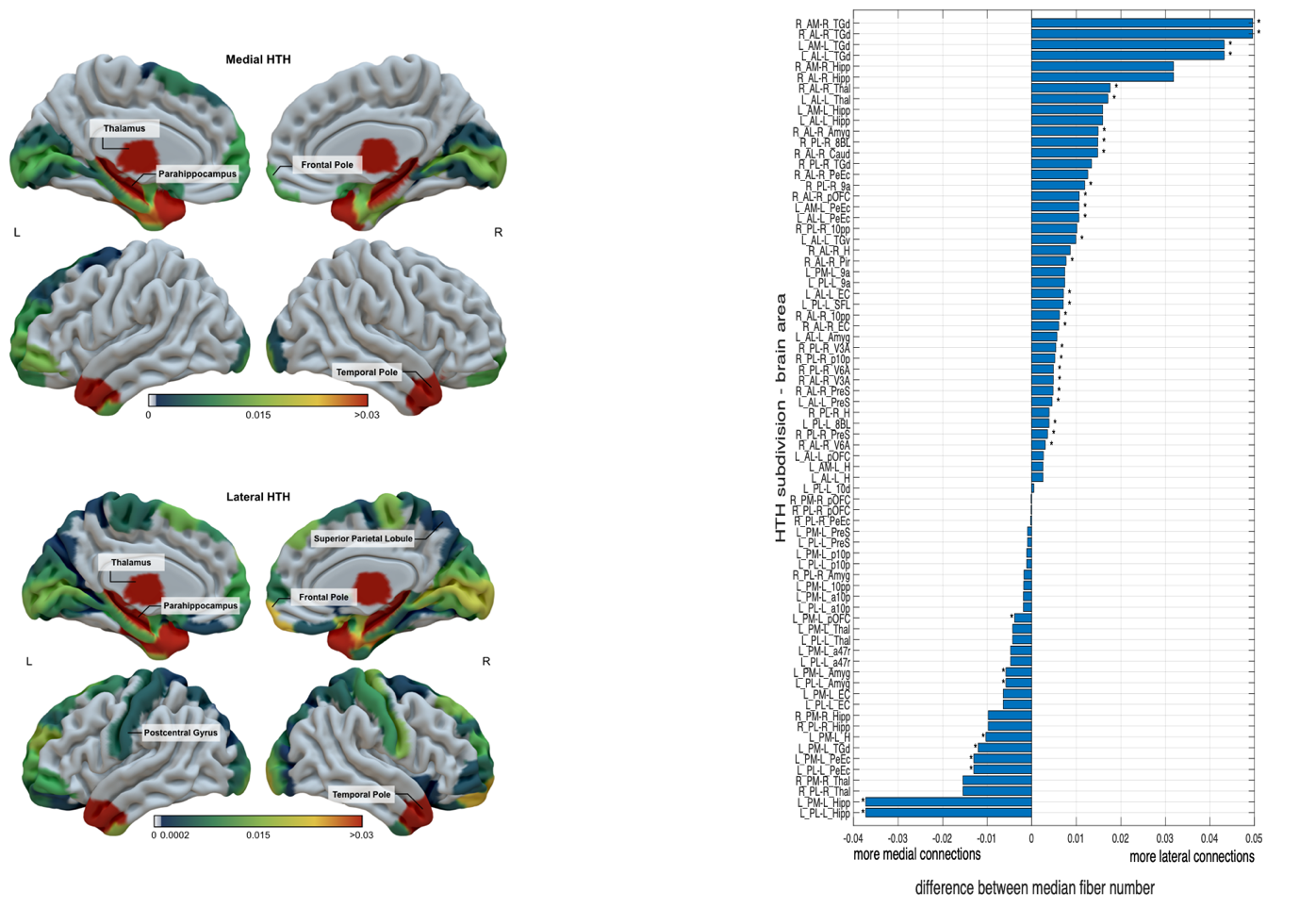

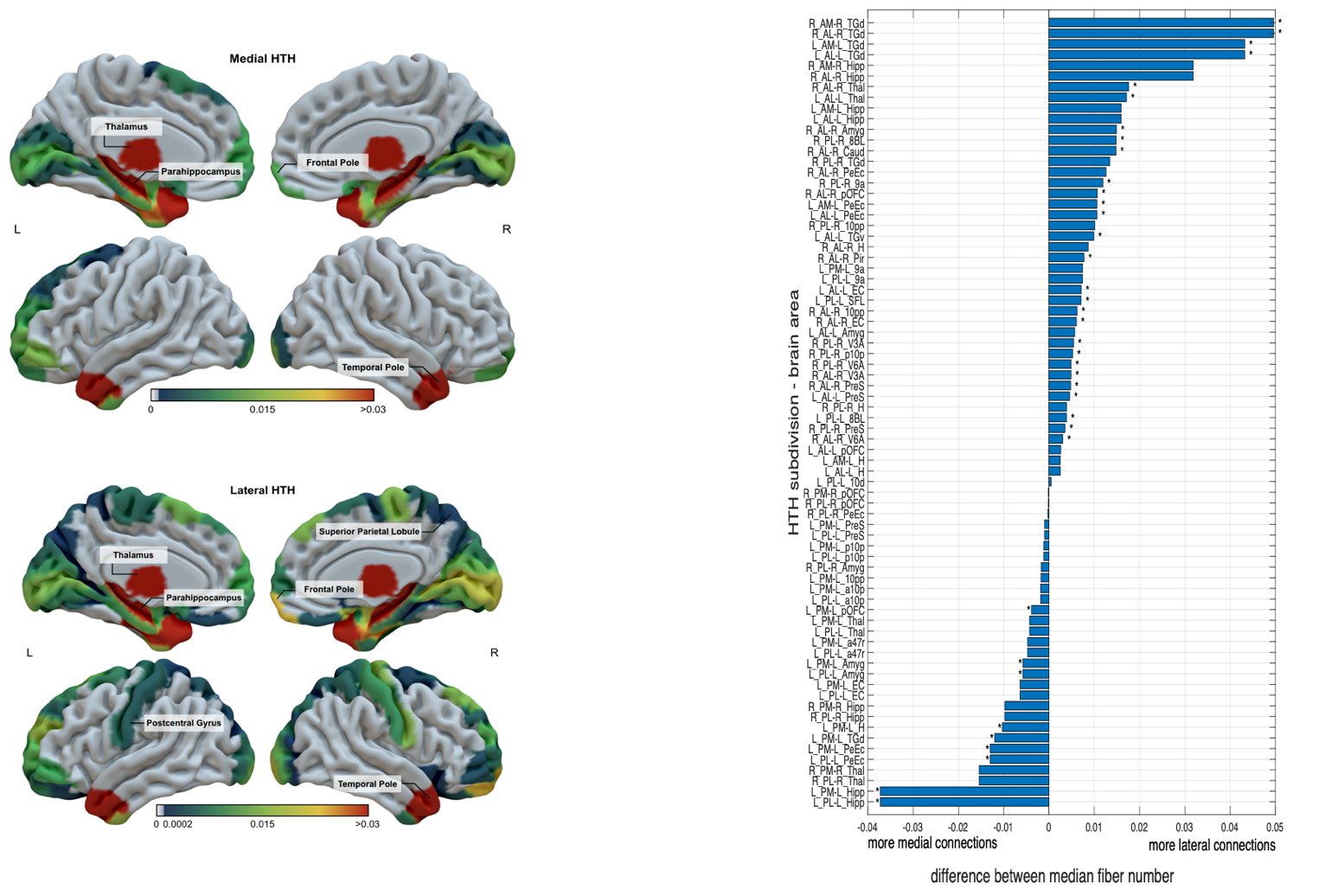


**Upper left:** structural connectivity of medial HTH.

**Lower left:** structural connectivity of lateral HTH.

We tracked the fiber numbers for each of the 8 subdivisions of HTH. Here, we used the max connectivity between the anterior lateral HTH connectivity and the posterior lateral HTH connectivity for the lateral HTH connectivity. The fiber numbers (normalized) with the left HTH were shown on the left hemisphere, while the connections with the right HTH were shown on the right hemisphere. Similarly, the connections with the medial HTH were shown in this figure.

**Right**: Differences between the lateral and the medial HTH connections, i.e. the difference between the medians of the fiber number tracked for the lateral and the medial HTH. The significance of such difference was assessed by a nonparametric test (i.e. the sign test). After controlling for multiple comparisons among the connections between 8 HTH subdivisions and 374 brain regions by false discovery rate, the significant (FDR<0.05) lateral-medial differences were marked with ‘*’.

## Figure S4. Cross-lagged path analyses between frontopolar volume and BMI in IMAGEN.

**
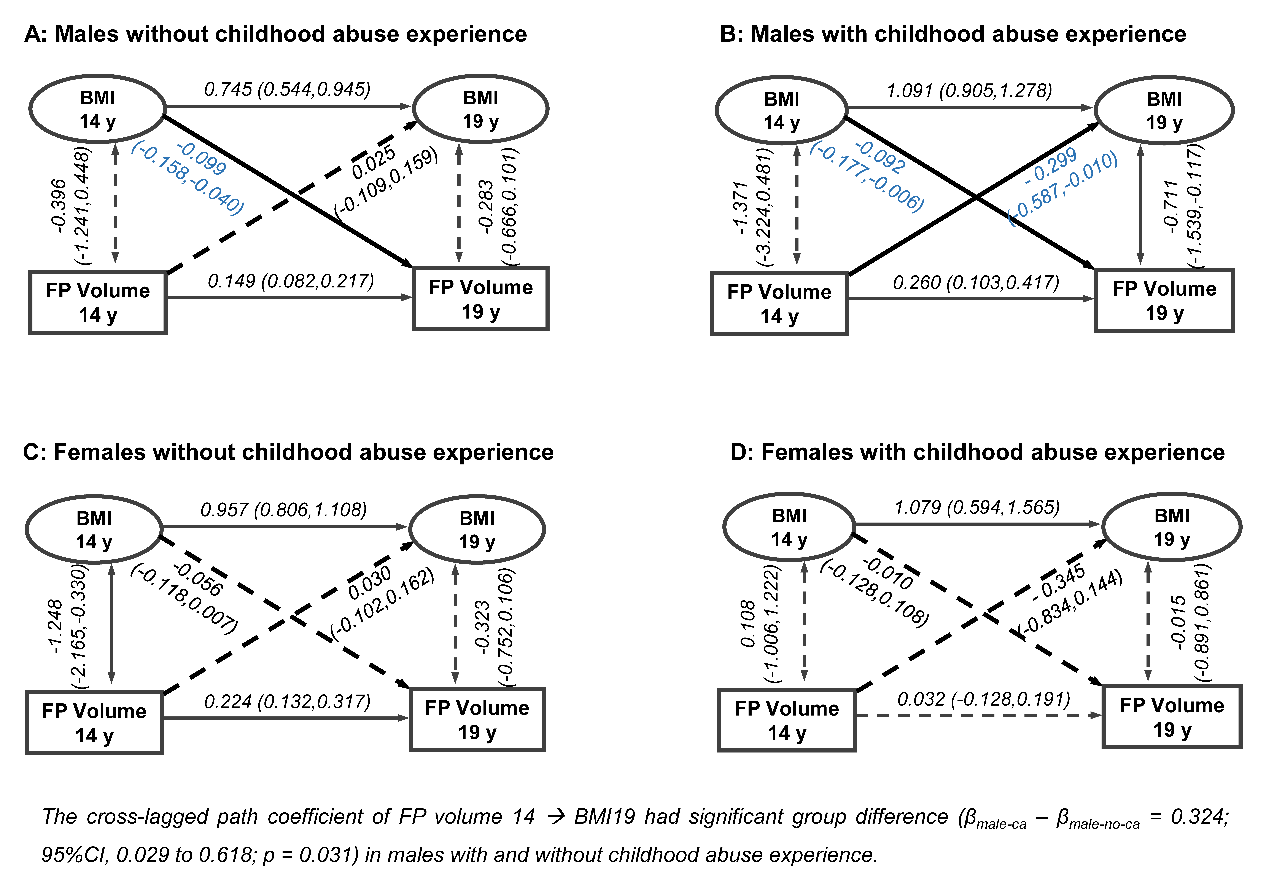
**

A: cross-lagged path analysis in males without any history of childhood abuse (n = 216). B: cross-lagged path analysis in males with a history of childhood abuse (n = 62). C: cross-lagged path analysis in females without any history of childhood abuse (n = 216). D: cross-lagged path analysis in females with a history of childhood abuse (n = 61). In both male groups, higher BMI at age 14 years was correlated with smaller FP at age 19 years (-.092/-.099, 95% CI -.176/-.156 to -.008/-.038; p=.04/.001; n=62/216).

# Appendix

**IMAGEN consortium author list** Lisa Albrecht (Charité), Chris Andrew (IoP), Mercedes Arroyo (Cambridge University), Eric Artiges (INSERM), Semiha Aydin (PTB), Christine Bach (Central Institute of Mental Health), Tobias Banaschewski (Central Institute of Mental Health), Alexis Barbot (Commissariat à l'Energie Atomique), Gareth Barker (IoP), Nathalie Boddaert (INSERM), Arun Bokde (Trinity College Dublin), Zuleima Bricaud (INSERM), Uli Bromberg (University of Hamburg), Ruediger Bruehl (PTB), Christian Büchel (University of Hamburg), Arnaud Cachia (INSERM), Anna Cattrell (IoP), Patricia Conrod (IoP), Patrick Constant (PERTIMM), Jeffrey Dalley (Cambridge University), Benjamin Decideur (Commissariat à l'Energie Atomique), Sylvane Desrivieres (IoP), Tahmine Fadai (University of Hamburg), Herta Flor (Central Institute of Mental Health), Vincent Frouin (Commissariat à l'Energie Atomique), Jürgen Gallinat (Charité), Hugh Garavan (Trinity College Dublin), Fanny Gollier Briand (INSERM), Penny Gowland (University of Nottingham), Bert Heinrichs (Deutsches Referenzzentrum für Ethik), Andreas Heinz (Charité), Nadja Heym (University of Nottingham), Thomas Hübner (Technische Universität Dresden), James Ireland (Delosis), Bernd Ittermann (PTB), Tianye Jia (IoP), Mark Lathrop (CNG), Dirk Lanzerath (Deutsches Referenzzentrum für Ethik), Claire Lawrence (U Nottingham), Hervé Lemaitre (INSERM), Katharina Lüdemann (Charité), Christine Macare (IoP), Catherine Mallik (IoP), Jean-François Mangin (INSERM), Karl Mann (Central Institute of Mental Health), JeanLuc Martinot (INSERM), Eva Mennigen (Technische Universität Dresden ), Fabiana Mesquita de Carvahlo (IoP), Xavier Mignon (PERTIMM), Ruben Miranda (INSERM), Kathrin Müller (Technische Universität Dresden), Frauke Nees (Central Institute of Mental Health), Charlotte Nymberg (IoP), Marie-Laure Paillere (INSERM), Tomas Paus (University of Toronto), Zdenka Pausova (University of Toronto), Jean-Baptiste Poline (Commissariat à l'Energie Atomique), Luise Poustka (Central Institute of Mental Health), Michael Rapp (Charité), Gabriel Robert (IoP), Jan Reuter (Charité), Marcella Rietschel (Central Institute of Mental Health), Stephan Ripke (Technische Universität Dresden), Trevor Robbins (Cambridge University), Sarah Rodehacke (Technische Universität Dresden), John Rogers (Delosis), Alexander Romanowski (Charité), Barbara Ruggeri (IoP), Christine Schmäl (Central Institute of Mental Health), Dirk Schmidt (Technische Universität Dresden), Sophia Schneider (University of Hamburg), MarkGunter Schumann (IoP), Florian Schubert (PTB), Yannick Schwartz (Commissariat à l'Energie Atomique), Michael Smolka (Technische Universität Dresden), Wolfgang Sommer (Central Institute of Mental Health), Rainer Spanagel (Central Institute of Mental Health), Claudia Speiser (GABO:milliarium mbH & Co. KG), Tade Spranger (Deutsches Referenzzentrum für Ethik / Institut of Science and Ethics), Alicia Stedman (University of Nottingham), Sabina Steiner (Central Institute of Mental Health), Dai Stephens (University of Sussex), Nicole Strache (Charité), Andreas Ströhle (Charité), Maren Struve (Central Institute of Mental Health), Naresh Subramaniam (Cambridge University), Lauren Topper (IoP), Walter (Charité), Robert Whelan (University College Dublin), Steve Williams (IoP), Juliana Yacubian (University of Hamburg), Monica Zilbovicius (INSERM), C Peng Wong (IoP), Steven Lubbe (IoP), Lourdes Martinez-Medina (IoP), Alinda Fernandes (IoP), Amir Tahmasebi (University of Toronto).
